# International Medical Congress

**Published:** 1868-01-01

**Authors:** 


### INTERNATIONAL MEDICAL CONGRESS,*

CONVENED AT PARIS, AUGUST 16, 1867.

TRANSLATED EXPRESSLY FOR THE JOURNAL BY PROF. FREER.

THE banquet was largely attended, but an air of sadness prevailed. The death of Velpeau — the news of which had been received too late to render it possible to delay this fête — gave an expression of mourning to the entire assemblage.

M. Bouillaud drank to the First International Medical Congress.

M. Palusciano drank to the health of President Bouillaud; which was highly applauded, and with justice.

M. Bouillaud had entered into the necessary work for the success of the Congress with his whole soul. One could see from the first that he deplored the restrictions that the programme — much too minute — imposed upon the discussions.

We reproduce the speeches of M. Bouillaud and M. Palusciano:

*M. Bouillaud.* GENTLEMEN: — What finer spectacle than the sessions of this assembly! It recalls, in spite of us, that which Cynéas saw when he took the Roman senate for an assemblage of kings. What saw then — said the master of French eloquence (J. J. Rousseau) — what saw then Cynéas that was so majestic? He saw the most beautiful spectacle that had ever appeared under the heavens: the assemblage of two hundred virtuous men, worthy to command Rome and the earth.

The spectacle of our medical senate is not thus sublime. It is not that of the masters of the world; but it is an assembly of more than seven hundred physicians, worthy to serve as models for all assemblies of savans and of sages. Salute, then, our first International Medical Congress.

* Continued from the December Number.

*M. Palusciano.*—It is not permitted to ourselves to appreciate the work that we will have achieved. The world and posterity will judge. But that which we can verify, is that the idea of a reunion of medical men of all nations for the purpose of discussion is a veritable progress. Our programme has been followed with exactness and fidelity, due to the lively interest and eminent qualities displayed by our honorable President.

In the medical world, no one ignores the important progress for which science is indebted to this learned professor of clinics of the faculty of Paris. And the most of us already know the biography of this indefatigable laborer, who is the honor of the medical faculty of France.

We wish, then, that all future medical congresses may be presided over by like veterans of progress and liberty; and in order to thank him, let each repeat, in his own proper tongue, *Vive Bouillaud!*

### COMPLEMENTARY SESSIONS.

At a complementary session on Thursday evening, the 22nd August, M. Brunetti exposed his new method of

### PRESERVING ANATOMICAL SPECIMENS.

His method comprises many complicated operations, comprising washing, removing the fat, tanning, and dessication.

The pieces are washed by passing pure water through the blood vessels and different excretory ducts; then the water is removed by passing alcohol through the same channels; following this with ether, in order to the removal of the fat. Having arrived at this point, the piece may be plunged in ether and kept an indefinite time before subjecting it to the subsequent processes, or tanning.

This is accomplished by dissolving tannic acid in boiling distilled water, a saturated solution, and by injecting the solution in the same manner as above, after having chased out the ether by means of injections of distilled water.

The final operation is that of drying the piece, which is attained by passing a heated atmosphere through the vessels and ducts of glands.

The specimens are flexible, light, retain their volume and natural relations. The histological elements are solid, for the liquids have been replaced by the tannic. These pieces can be handled without fear of injury, and may be preserved indefinitely.

After M. Brunetti, M. Laskanski presented some anatomical pieces which were wonderfully preserved, and which had the advantage of retaining the natural aspect respecting suppleness and all other qualities of normal tissues. Unfortunately, M. Laskanski was not prepared to make a revelation of his method of preparation, merely divulging that *Phenic acid* constituted a part of the liquid used in making the injection for the vessels.

The complementary sessions being destined for communications foreign to the primitive programme, were occupied by subjects that had had no relation to each other. Some of them were of real importance. We will speak of that on the subject of the

### PROPAGATION OF CHOLERA.

M. Shrimpton observed that the characteristic and constant symptoms of cholera were: 1st. Abnormal cold. 2nd. Alteration of the respiration and non-oxygenation of the blood. 3rd. The intestinal flux. According to him, all the primary phenomena are explained by the asphyxie of the elementary tissues. As soon as the choleraic influences have ceased, reaction may occur even after decease, that which proves that this reaction and the morbid action which preceded have for their seat the cells, the life of which persists still a certain time after the termination of general life.

We can not follow M. Shrimpton in the development of his theory, only adding that he supports the non-contagion theory.

The convictions of M. Shrimpton upon this subject are very firm, as we can see by the following extract:

"Cholera can not be contagious, because the choleric can not carry on themselves the germs of the contagion. To carry the germs of contagion, supposes a labor of elaboration, a period of incubation, such as take place in acknowledged contagious maladies, such as the typhus, yellow, and eruptive fevers. Now this can not take place with those affected with cholera, for as soon as patients are affected with cholera influences, all organic action ceasing, the choleric influence must necessarily extinguish itself in each person, whatever may be their number. Respecting myself, I ask, for instance, that our illustrious President, M. Bouillaud, and our learned Vice-President, M. Ricord, conjointly with my honorable colleague of the Hospital Gagliani, may form a commission, to which they may add a delegate from each foreign country, now in Paris, to submit me to all the proofs they may desire to indicate. I will go to bed with a person in the active stage of cholera, will respire his breath as long as the commission may deem it necessary; I will inoculate myself with all the matter proceeding from the body of the patient; in a word, I will submit myself under their eyes to all that may be demanded to carry conviction of the non-contagiousness of cholera to the entire world."

*M. Crocq.*—"If I speak, it is not for the purpose of opening the question of the nature of cholera, which would require too much time. Notwithstanding, I must say that in most cases, if not in all, the abdominal phenomena designated as premonitory precede the phenomena of asphyxie. These latter constitute neither the essence nor the point of departure of the malady.

"I but throw out this remark, in passing, the time not permitting us to attack this important question.

"My object in speaking is to protest against the doctrines of M. Shrimpton relatively to its contagion. It is very easy to declare that cholera is not contagious; that one must not fear it; that one may confront its contact with impunity; but it is necessary, above all things, to know the truth, even when it may be disagreeable. Besides, know well the enemy that one has to face, and avow the fact of contagion if it possesses it, than to dissimulate. Fear not that the choleric patients will lack attendance because physicians may recognize the contagiousness of their disease. Do we not care for other contagious and infectious diseases, at the time recognizing their transmissibility? No, gentlemen, the true physician does not recoil before contagious maladies; he knows that which his obligations command, and does not fail to execute.

"Be not astonished, then, to hear me proclaim the contagiousness of cholera. Numberless observations have convinced me of this. M. Shrimpton has informed us that he is ready to inoculate himself with the blood or any fluids whatever of the *cholóriques.* That amounts to nothing with me as a contagionist; I am ready to submit to the same ordeal; and I am sure it would contribute to valuable results in the enlightenment respecting the problem of contagion or non-contagion."

